# Novel Biobased Epoxy Thermosets and Coatings from Poly(limonene carbonate) Oxide and Synthetic Hardeners

**DOI:** 10.1021/acssuschemeng.1c07665

**Published:** 2022-02-18

**Authors:** Vitor Bonamigo Moreira, Jeroen Rintjema, Fernando Bravo, Arjan W. Kleij, Lourdes Franco, Jordi Puiggalí, Carlos Alemán, Elaine Armelin

**Affiliations:** † Departament d’Enginyeria Química, Universitat Politècnica de Catalunya (UPC), Campus Diagonal Besòs (EEBE), C/Eduard Maristany, 10-14, Building I, 2nd Floor, 08019 Barcelona, Spain; ‡ Programa de Pós-graduação em Engenharias de Minas, Metalúrgica e de Materiais (PPGE3M), Universidade Federal do Rio Grande do Sul (UFRGS), Av. Bento Gonçalves, 9500, Porto Alegre, 91501-970 Rio Grande do Sul, Brazil; § Barcelona Research Center for Multiscale Science and Engineering, Universitat Politècnica de Catalunya (UPC), Campus Diagonal Besòs (EEBE), C/Eduard Maristany, 10-14, Building I, Basement Floor, 08019 Barcelona, Spain; ∥ Institute of Chemical Research of Catalonia (ICIQ), The Barcelona Institute of Science and Technology, Av. Països Catalans 16, 43007 Tarragona, Spain; ⊥ Catalan Institute of Research and Advanced Studies (ICREA), Pg. Lluis Companys 23, 08010 Barcelona, Spain; # Institute for Bioengineering of Catalonia (IBEC), The Barcelona Institute of Science and Technology, Baldiri Reixac 10-12, 08028 Barcelona, Spain

**Keywords:** poly(limonene carbonate) oxide, epoxy thermoset, thermal properties, mechanical properties, solvent-free paint

## Abstract

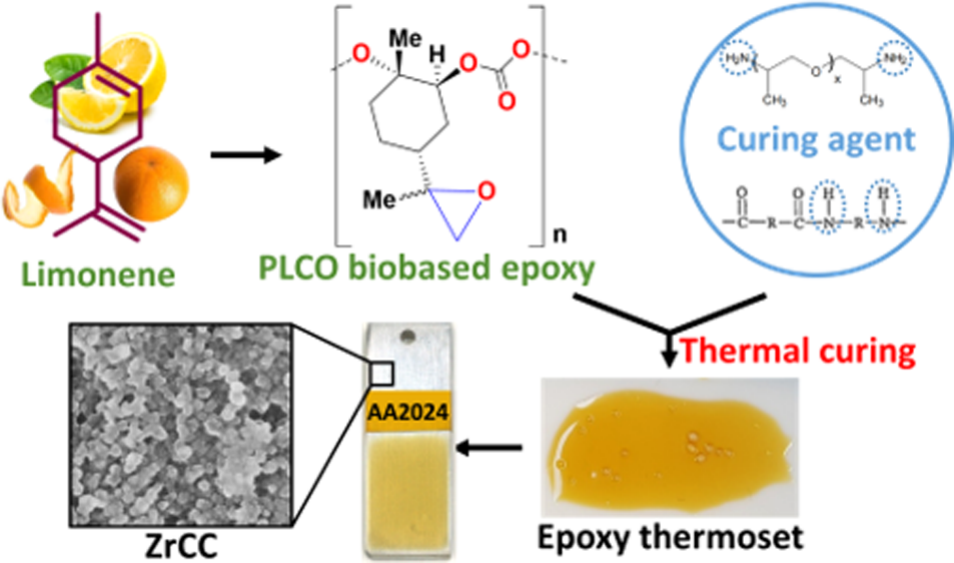

In the area of coating development, it is extremely difficult to find a substitute for bisphenol A diglycidyl ether (DGEBA), the classical petroleum-based raw material used for the formulation of epoxy thermosets. This epoxy resin offers fast curing reaction with several hardeners and the best thermal and chemical resistance properties for applications in coatings and adhesive technologies. In this work, a new biobased epoxy, derived from poly(limonene carbonate) oxide (PLCO), was combined with polyetheramine and polyamineamide curing agents, offering a spectrum of thermal and mechanical properties, superior to DGEBA-based thermosets. The best formulation was found to be a combination of PLCO and a commercial curing agent (Jeffamine) in a stoichiometric 1:1 ratio. Although PLCO is a solid due to its high molecular weight, it was possible to create a two-component partially biobased epoxy paint without the need of volatile organic compounds (i.e., solvent-free formulation), intended for use in coating technology to partially replace DGEBA-based thermosets.

## Abstract 2

A sustainable epoxy thermoset from limonene derivatives for coatings applications is designed.

## Introduction

Biobased products generate increasing interest in coating and adhesive industries thanks to new performance advantages and added benefits of not relying on petroleum feedstock.^1−4^ Nowadays, there is an impressive portfolio of biobased compounds that will play an even larger role in materials technology with a huge variety of building blocks.^2,5^ Examples of biobased precursors include glycols, natural oils, and fatty acids used in resin production^6,7^ and agricultural byproducts such as starch and lignin compounds.^3,8−10^ However, in the quest to find and identify renewable raw materials for use in coatings and adhesives, epoxy materials are lagging behind compared to other polymers (e.g., biobased polyurethanes and polyesters)^5,11^ to be released as commercially available products. For instance, one-to-one replacement of petroleum-based products with epoxy biobased products is not yet possible.

Furthermore, ever since the establishment of volatile organic compound (VOC) regulations in Europe in 1999,^12,13^ the most important investigations have been devoted to the adaptation from epoxy solvent-borne formulations toward water-borne and solvent-free systems. However, the use of solvent-based epoxy paints cannot be completely banned from the market, basically due to their fast cross-linking reaction, high waterproof properties, or their reduced drying times, which represent important advantages for their applications. Some examples of epoxy solvent-borne systems are coil coatings, which require fast solvent evaporation and curing times (<1 min), and stoving enamels, which are paints that cure at elevated temperatures (80–250 °C).^14^ Consequently, researchers and developers, both in academia and in industry, are looking for combinations of fossil fuel primary compounds with renewable raw materials as realistic market alternatives. One example is the commercialization of biorenewable epichlorohydrin (ECH), one of the intermediates of bisphenol A diglycidyl ether (commonly abbreviated as DGEBA), in the production line of epoxy resins.^15^

The production of polymers using natural and sustainable raw materials has been widely studied with promising results.^2,3,5,6,16−19^ One well-known, low-cost sustainable raw material is limonene, which is extracted from citrus fruit peels and can be used as a monomer in the production of several polymers and blends.^20,21^ One promising example is the work developed by Mija et al.,^22^ who used limonene dioxide and glutaric anhydride to obtain fully biobased thermoset materials. Moreover, polycarbonates and crosslinked polymers are other alternatives with potential applications in the coating and adhesive industry.^23−26^ In this regard, poly(limonene carbonate)s (PLCs), obtained from limonene and carbon dioxide, through well-developed metal catalysis processes, are emerging as potential candidates to replace petroleum-based polymers.^5,25,27,28^ The alkene pendant groups in PLC can be easily oxidized in high yield to provide synthetically versatile epoxide groups without alteration of the polycarbonate backbone linkages.^25,28^ This epoxy-based polymer, poly(limonene carbonate oxide) (PLCO) , can be scaled up to multi-gram quantities that are useful in the context of pilot market studies. The synthesis of PLCO from PLC was first reported by Kleij and co-workers,^25^ resulting in high-yield and controllable-molecular-weight epoxy systems. PLCO has been explored in this study to produce thermosets by curing with four commercially available polyamines as reactive hardeners.

The principal aim of this work is to demonstrate the use of a readily available biobased epoxy thermoset in coating technology that would enable the transition of suitably mature technologies from a purely academic to a commercially applied level. The results herein describe the film processing; the thermal, mechanical, and permeability properties; and the application potential of the new thermoset materials in a solvent-free paint formulation. According to the chemical nature of the hardener and the molar ratio of components A (epoxy resin) and B (curing agent), it is possible to modulate the thermal and the mechanical properties of the thermoset films.

## Experimental Section

### Materials

PLC and PLCO were prepared as previously described, and the molecular weight was not noticeably affected by the epoxidation of the pendent double bonds.^25^ The final PLCO had a *M*_n_ of 6.0–7.0 kg/mol, a *M*_w_ of 8.0–9.0 kg/mol, and a Đ of 1.30–1.34 (see two examples of the gel permeation chromatography analysis in the Supporting Information, Figure S1). This PLCO sample showed a *T*_g_ of 126 °C and has a *T*_d_^5%^ of 233 °C. The epoxy-equivalent weight of PLCO varied depending on each preparation (EEW = 216–315 g/equiv). It was determined by titration of PLCO with KOH (ASTM D1652) and corresponds to an approximate 97–98% epoxidation of the double bonds present in PLC. We should mention that several batches of products were used along the project. An EEW of 216 g/equiv was chosen for the calculations of the stoichiometric and off-stoichiometric compositions.

Diglycidyl ether of bisphenol A (DGEBA, Sigma-Aldrich, *M*_w_ 340.4 g/mol, EEW 172–176 g/equiv) was used as received. In this case, an EEW of 172 g/equiv was chosen for the calculations of hardener amount. Diethylenetriamine (DETA, Sigma-Aldrich, AHEW 21 g/equiv), branched polyethylenimine (PEI, Lupasol PR 8515, BASF SE, *M*_w_ 2000 g/mol, AHEW 37 g/equiv), polyoxypropylenediamine (Jeffamine D-400, Huntsman Corp., AHEW 115 g/equiv), and Crayamid 195 × 60 (Arkema Coating Resins, AHEW 240 g/equiv) were used as hardeners, and 1-methylimidazole (1-MI, Sigma-Aldrich) was used as an anionic initiator. Solvents used in the present study were all supplied from PanReac Chemical Spain, which are of analytical grade. For the high-solid epoxy formulation, the following materials were employed: benzyl alcohol (ReagentPlus, Sigma-Aldrich Corporation), titanium dioxide (OXINED BLANCO, Euro Pigments), defoamer/air release agent (BYK-A 530, BYK Additives & Instruments), and silica nanoparticles (Si-NPs) prepared following the procedure described elsewhere by Stöber synthesis and the microemulsion method.^29,30^ Aluminum sheets (AA2024 alloy, 5.0 × 1.5 × 0.3 cm^3^) were used as substrates for electrochemical impedance spectroscopy (EIS) tests. Saloclean 667N (Klintex Insumos Industriais Ltd.) was the degreasing agent used for the pre-treatment of aluminum sheets.

### Epoxy Film Preparation and Stoichiometry

The compositions of PLCO:hardener were defined taking into account the epoxy equivalent weight (EEW) of PLCO (216 g/equiv) and the amine hydrogen equivalent weight of the hardeners. As the curing agents DETA and PEI resulted in bad quality films, the molar ratio described here refers only to Jeff and Cray (AHEW: 115 g/equiv for Jeffamine and 240 g/equiv for Crayamid). For the formulations containing DGEBA, the composition followed the same stoichiometric proportion approach and an EEW of 172 g/equiv. Samples were prepared using the stoichiometric and off-stoichiometric proportions of 2:1, 1:1, and 1:2 for PLCO:hardener, that is, the theoretically necessary amount of both components in order to have each epoxy group reacting with one amine functionality. The amount of 1-MI compound (a well-known initiator molecule for the ring-opening aminolysis of epoxies)^31−33^ was constant for all formulations in which it was tested (2% by weight) and ensured efficient conversion of the sterically protected epoxy groups in PLCO. Table S1 (Supporting Information) summarizes the main properties of the raw materials used in this work.

### Thermoset Curing Protocol

PLCO (component A, 100 mg), which is a fine yellowish powder, was initially dissolved in a small proportion of xylene (50 μL), and after dissolution, it was mixed with the necessary amount of the hardener (component B) (Table S2). No solvent was needed for the curing amines because they are all viscous liquids. Components A and B (and the initiator, when used) were vigorously stirred at room temperature. The mixture was then poured into a glass Petri dish, covered by Teflon films, and left overnight in a ventilation hood for solvent evaporation. Before the Fourier-transform infrared spectroscopy (FTIR) study and the differential scanning calorimetry (DSC) measurements, the samples were pre-cured under vacuum for 2 h at 120 °C in order to activate the initial crosslinking process and remove the entire residual hydrocarbon solvent.

### Characterization Techniques

**Scheme 1:**
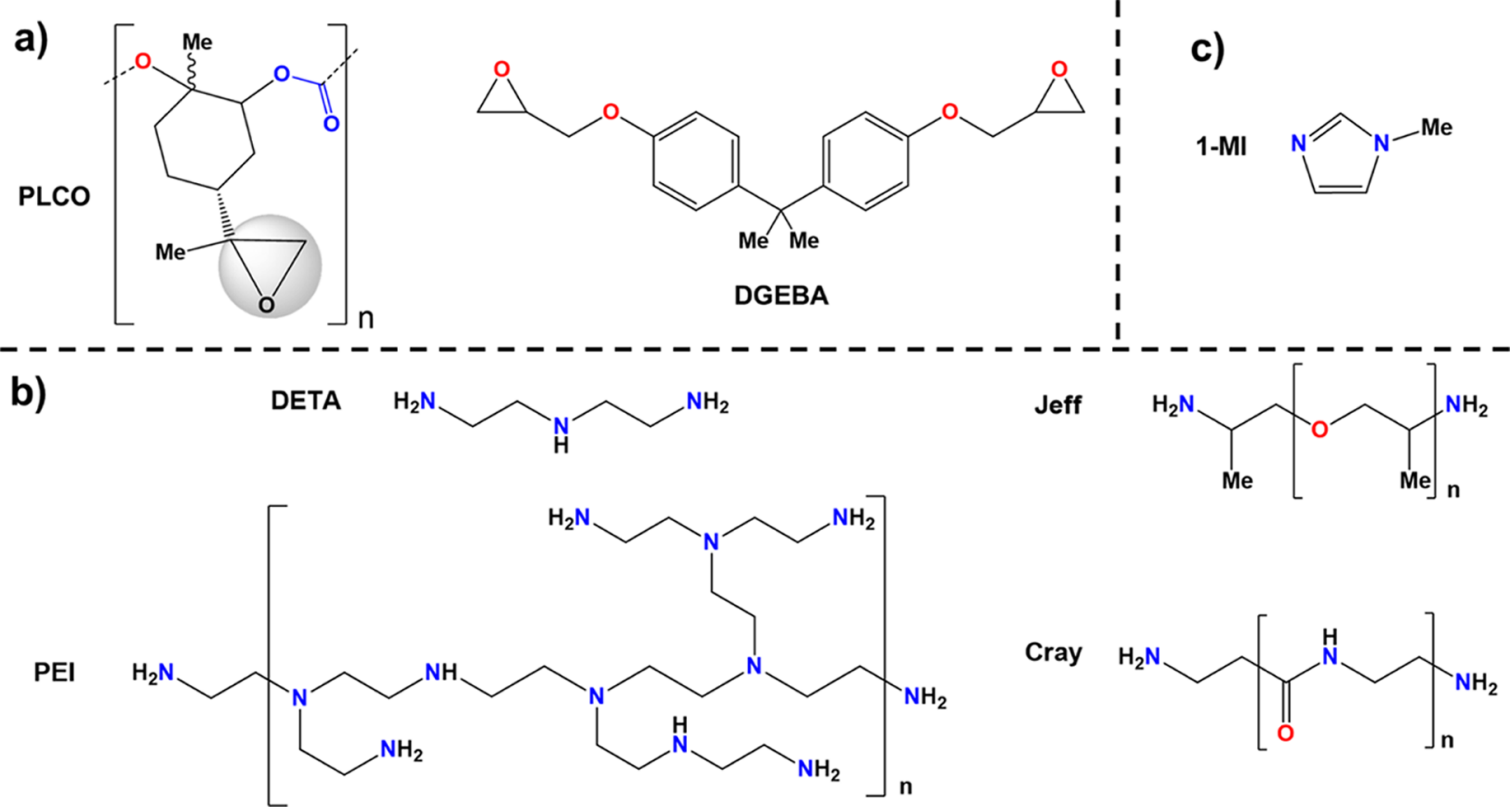
Structures of Compounds Used for the Epoxy Thermoset Formulations: (a) Epoxy Resins, (b) Curing Agents, and (c) Initiator Molecules

Thermoset chemical composition was evaluated with FTIR. A Jasco 4100 spectrophotometer, coupled with an attenuated total reflection (ATR) accessory (Specac model MKII Golden Gate Heated Single Reflection Diamond ATR), allowed the monitoring of the crosslinking reactions between component A (epoxies, Scheme 1a) and component B (curing agents, Scheme 1b). Cured thermoset films were characterized by isothermal FTIR–ATR, compared with the raw materials and −NH–CH_2_–C(OH)– formation. The resolution used was 8 cm^–1^, the number of accumulated scans for each sample was 32, and the wavenumber range was from 600 to 4000 cm^–1^.

Dynamic DSC tests were carried out with the pre-cured samples in order to assess the final curing temperature for each composition, identified by an exothermal peak in the first thermal sweep of the specimens. The kinetics of the curing has been evaluated by calorimetry using a TA Instruments Q100 series equipped with a refrigerated cooling system and operating under a nitrogen atmosphere. First, the films were pre-cured isothermally at 120 °C for 2 h. After that, two dynamic scans were performed from −90 to 200–250 °C (depending on the stability of the samples) at 10 °C/min.

For the isothermal experiments, the protocol consisted of heating the pre-cured samples (2 h at 120 °C) before moving them to the calorimeter and further post-curing at high temperature to start the isothermal assay. Once this temperature was reached (165 °C for compositions with Jeff and 160 °C for Cray, respectively), it was maintained for 2 h in order to promote the isothermal curing. After 2 h of isothermal curing, the samples were cooled to −80 °C and heated at 10 °C/min until sample degradation was observed. The integration of the observed curing peak during the isothermal curing step provided the curing degree of the studied compositions. The total heat of curing can be measured in the isothermal curing step carried out in the calorimeter. The degree of epoxy conversion (%) was calculated from the isothermal DSC curves, following eq 1
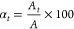
1where α_*t*_ is the degree of epoxy conversion, in %, at time *t*, *A*_*t*_ is the area of the curing peak at time *t*, and *A* is the area of the entire curing peak after full curing is achieved.

*T*_g_s were determined at the half-way point of the jump in the second heating curve (UNE-EN-ISO 11357-2) after complete curing of the samples (i.e., the absence of a residual exotherm). The values correspond to the ultimate *T*_g_ (*T*_g∞_).

Thermogravimetric analysis (TGA) was performed under a nitrogen atmosphere with Q50 (TA Instruments) equipment at 10° C/min in the range between 30 and 600 °C. This test was carried out with fully cured samples to evaluate the thermal stability of the thermosets. The results were expressed as *T*_d_^5%^ and *T*_d,max_, corresponding to the initial degradation temperature of 5 wt % weight loss and the highest temperatures of polymer backbone decomposition observed, respectively.

Determination of gel content was carried out on the cured films by measuring the weight loss after 24 h of swelling in the xylene solvent at 80 °C by using test method C described in the ASTM D2765-16 standard. The results are expressed as follows
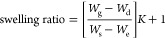
2
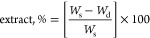
3where *W*_g_ is the weight of the swollen polymer after the immersion period; *W*_d_ is the weight of the dried polymer; *W*_s_ is the weight of the specimen being tested; *W*_e_ is the weight of the extract (amount of polymer extracted from the specimen in the test), that is *W*_e_ = *W*_s_ – *W*_d_; and *K* is the ratio of the density of the polymer to that of the solvent at the immersion temperature.

The mechanical properties were evaluated at room temperature using a universal testing machine (Zwick BZ2.5/TN1S) with specially designed grips. The specimens were cut in a rectangular shape of 30 × 3 mm^2^ and variable thicknesses. The experiments were performed at a crosshead speed of 5 mm/min and with a pre-load of 0.05 MPa (Table S3).

In order to assess the polymer permeability as a primer, samples for EIS tests were prepared using AA2024 alloy substrates. These substrates were polished to #2500 grit and went through an alkaline degreasing procedure (pH 9.4, 70 g/L, 70 °C) during 5 min, followed by rinsing with deionized water and drying. Afterward, a nanometric layer of ZrO_2_ was applied in each sample to create a passivating layer for further polymer anchoring. The complete procedure was recently published in our previous work.^34,35^ The polymer film was applied on the metallic substrates by using pipettes and followed the abovementioned curing procedure. The dry film thickness was 238 ± 20 μm.

The EIS experiments were carried out using an Autolab PGSTAT302N potentiostat/galvanostat (Ecochemie). A three-electrode cell configuration was used, with a Ag|AgCl (KCl, 3 M) reference electrode and a platinum counter electrode with 0.05 M NaCl as the electrolyte. The coated aluminum sheet was the working electrode in this setup. An area of 0.785 cm^2^ was used for the measurements. After 30 min of open-circuit potential stabilization, an alternate potential with a 10 mV amplitude was applied in frequencies ranging from 10^5^ to 10^–1^ Hz, with 10 measurements per decade in logarithmic distribution. The measurements were performed after specific periods of exposure of the sample to the electrolyte, which were 1, 3, 5, 9, 12, and 15 h.

The experimental data were fitted with the simplified Randles circuit (*R*_s_[*R*_c_·CPE_c_]) to achieve the electrical equivalent-circuit (EEC) parameters expressed in Table S4.

### Preparation of the Solvent-Free Two-Component Epoxy Paint Formulation and Its Characterization

To obtain the initial paste, 8.46 g of PLCO (solid) was mixed with 33.82 g of DGEBA (liquid), without a solvent. This proportion was calculated to have 20% of biobased epoxy respect to 80% of the synthetic one. Afterward, 5.01 g of TiO_2_ (white pigment) and 48.67 g of silica nanoparticles (fine powder used as a filler) were added, followed by 2.04 mL of BYK A530 (liquid defoamer) and 2.08 mL of benzyl alcohol (solvent). This corresponds to component A (partially biobased epoxy resin). Then, the mixture was milled in a mortar until a homogeneous and consistent paste was obtained. Afterward, 27.89 mL of component B (Jeffamine hardener) was added and left to react with component A for 30 min under hand-mixing. After that, the paste was applied to a Teflon substrate to provide films for physical–chemical evaluation. The films were post-cured in an oven at 150 °C for 2 h to ensure the complete curing of the lower-reactive epoxy groups present in PLCO. Table S5 summarizes the wt % of all components of the as-prepared paste composition.

Infrared characterization and TGA characterization were performed with the same equipment and procedures described. Table S5 shows the chemical formulation in weight percentage (wt %).

## Results and Discussion

### Biobased Epoxy Thermoset Preparations

**Figure 1:**
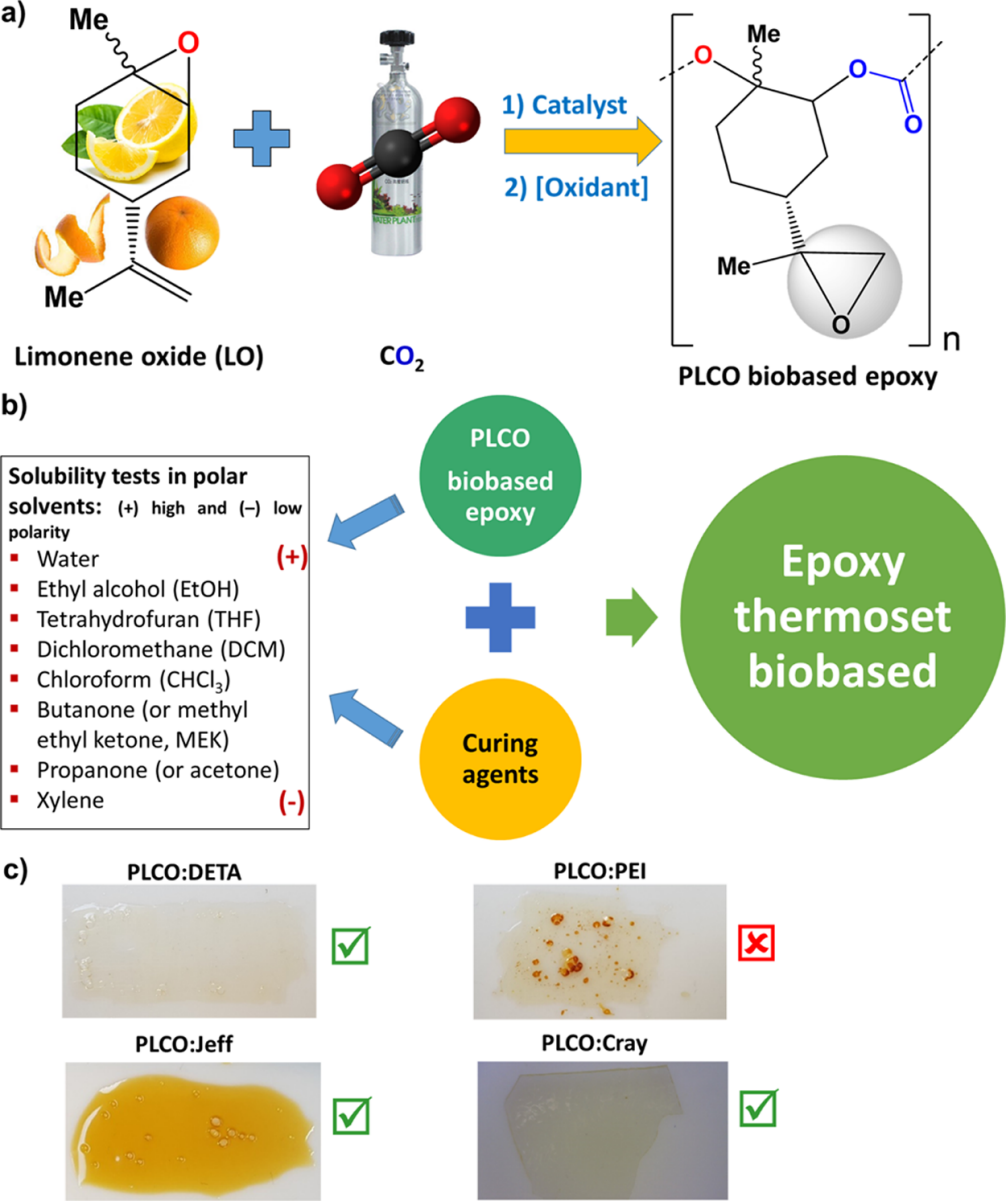
(a) Simplified route for obtaining PLCO biobased epoxy; (b) solubility test of PLCO and curing agents with polar solvents for the preparation of the thermoset polymer; (c) aspect of the films after curing: PLCO:DETA (brittle), PLCO:PEI (immiscible oligomers), PLCO:Jeff (homogeneous and with mechanical integrity), and PLCO:Cray (homogeneous and with mechanical integrity). Films were prepared using xylene as a solvent.

As the objective set out in our work is to convey the message that biobased epoxy may partially replace conventional, fossil-fuel-based epoxies in some applications, the following study was carried out by comparing some properties of the new materials derived from PLCO (yellow powder) to those prepared from the classical reagent DGEBA (viscous liquid) (Scheme 1a). The oxirane groups from DGEBA exhibit very high reactivity, being capable of reacting with a variety of functional groups such as anhydrides, acids, urethanes, amines, and thiols.^36−38^ Frequently, amines and polyamines are chosen as commercial hardeners because they react at room temperature with DGEBA and offer thermoset films with good crosslinking degrees and high chemical resistance. Unfortunately, in previous works,^39,40^ the somewhat congested epoxy groups from oxidized limonene (representing a subunit within the PLCO pre-polymer) were not found to be as reactive as DGEBA, requiring thus longer reaction times, higher temperatures, and post-curing treatment. According to Soto and Koschek,^40^ terminal epoxides are more reactive than endocyclic ones. However, the label mobility of the cyclohexane ring in limonene dioxides, with the state transitions between “chair-like” and “boat-like”, also leads to a decrease in the reactivity of the epoxide groups. Therefore, aromatic epoxides usually cure at room temperature with nucleophilic amines, whereas cycloaliphatic ones do not. Moreover, the molecular weight, the curing time, the curing degree, and the mechanical properties of the final thermoset PLCO-derived product will significantly depend on the hardener efficacy. The presence of an initiator molecule that can ensure high epoxy conversion levels is also envisaged in many cases. In our study, polyamines, polyimines, and polyamides (all commercially available) were chosen as benchmark and reactive curing agents to test the reactivity of the epoxy groups present in PLCO (Scheme 1b, Table S1). Furthermore, in order to increase the epoxy reactivity and accelerate the curing process, 1-methylimidazole (1-MI) was used as an initiator molecule (Scheme 1c). Regarding the biobased polymer, the synthetic route and reaction conditions to access controlled-molecular-weight PLCO (*M*_n_ ∼ 9000 g/mol) and polydispersity (Đ ∼ 1.2–1.3) were described elsewhere (Figure 1a).^25^ Additional data are included in the Supporting Information (Table S1 and Figure S1).

Before mixing components A (PLCO) and B (curing agents), the solubility of each one was tested considering the most important polar solvents used in chemistry and paint technology. Positive results were obtained in halogenated solvents, ketones, and aromatic hydrocarbons like xylene (Figures 1b and S2), with the latter and methyl ethyl ketone being the most extensively used solvent and co-solvent, respectively, in epoxy solvent-borne formulations. Xylene was finally chosen due to its relevance in the coating industry. The small amount of solvent used (0.5 mL/g of PLCO resin, Table S2) is compliant with VOC regulations.^12,13^ However, we do not discard the possibility to use other solvents in future works that comply with the Safety, Health and Environment (SH&E) criteria described elsewhere.^41^ As can be deduced from Figure 1c, good film forming properties were achieved with diethylenetriamine (DETA), polyoxypropylenediamine (Jeffamine D-400, hereafter abbreviated as Jeff), and polyamineamide (Crayamid 195 × 60, hereafter denoted as Cray) hardeners in the xylene solvent, whereas PLCO with PEI was not compatible. Despite the homogeneity and fast drying properties of the PLCO:DETA film, it was discarded for further assays due to its brittle behavior, attributed to the few methylene units of the curing agent. Finally, Jeff and Cray, representing commercially available and widely used curing agents, were selected to test the reactivity of the PLCO epoxy. Table S1 summarizes the main characteristics of the raw materials, and Table S2 shows the molar ratio and weight of each component in the thermoset formulation.

The compositions of epoxy:hardener were defined taking into account the EEW of each component (PLCO = 216 g/equiv; DGEBA = 172 g/equiv) and the amine hydrogen equivalent weight (AHEW) of the hardeners (Jeff = 115 g/equiv; Cray = 240 g/equiv). Samples were prepared using the proportions of 2:1, 1:1, and 1:2 of epoxy:hardener components in order to obtain stoichiometric (1:1) and sub-stoichiometric (2:1 and 1:2) amine–epoxy thermosets. PLCO is a finely divided and stable powder, easy to dissolve in small proportions of the solvent before mixing with the necessary mass of the curing agent, which are all liquids. These mixtures were left overnight in a ventilation hood for solvent evaporation. Before calorimetry measurements, the samples were pre-cured under vacuum for 2 h at 120 °C in order to activate the initial crosslinking process and remove the entire residual hydrocarbon solvent. However, the thermal property evaluation revealed that such PLCO epoxy needs temperatures higher than 120 °C for complete curing (data discussed in the next section).

**Figure 2:**
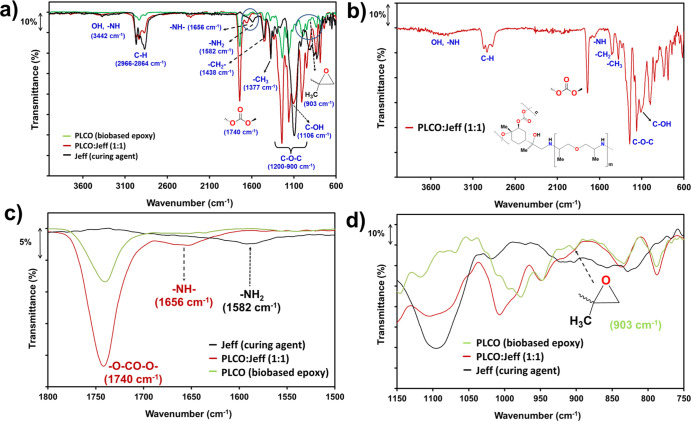
(a) FTIR spectrum of PLCO:Jeff (1:1) compared to PLCO biobased epoxy and the polyoxypropylenediamine (Jeff) curing agent; (b) PLCO:Jeff (1:1) thermoset main absorption bands; (c,d) amplified FTIR spectra of wavenumber ranges of 1800–1500 and 950–800 cm^–1^, showing, respectively, the disappearance of NH_2_ linkages and reduction of oxirane groups.

**Figure 3:**
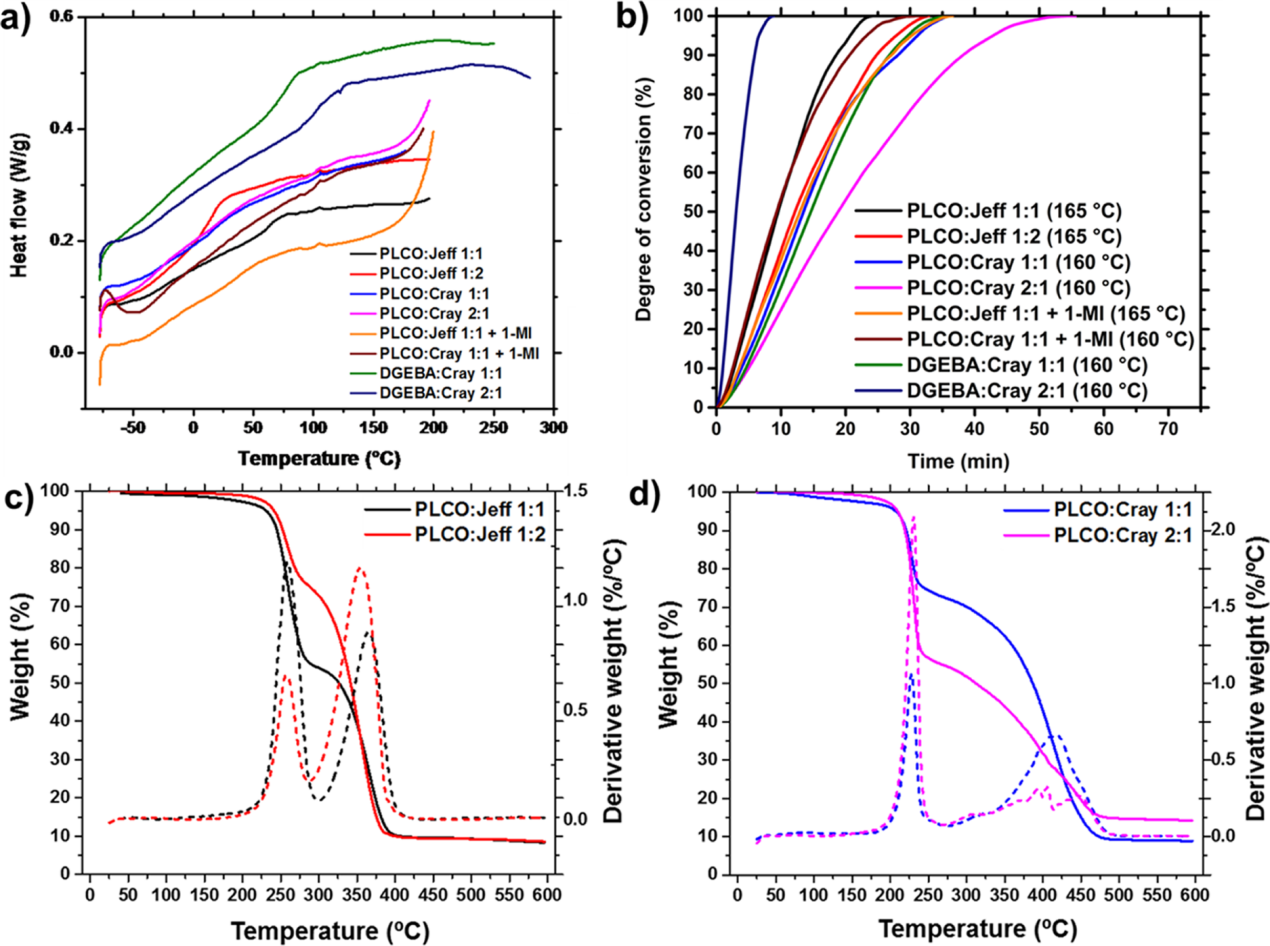
(a) Dynamic DSC curves corresponding to the second heating rate of the biobased thermosets investigated, compared to the petroleum-based classical epoxy (DGEBA); (b) degree of epoxide conversion (%) vs time (min) of all systems under isothermal calorimetry analysis, where the temperatures among parenthesis are that related to the post-curing process for each formulation; (c,d) thermogravimetric analysis of PLCO:Jeff (1:1 and 1:2) and PLCO:Cray (1:1 and 2:1), respectively.

The composition of the new partially biobased epoxy thermosets was examined by infrared spectroscopy (FTIR–ATR) and dynamic DSC (Figures 2 and 3). In a first approximation, the high number of polar groups of the new thermosets, originating from components A and B, complicates the straightforward analysis of the pendant oxirane groups due to the very low transmittance (903 cm^–1^, Figure 2a) in PLCO, when compared with that from DGEBA (917 cm^–1^, Figure S3). However, close inspection of the cured product, in comparison to the individual monomers, indicates that new absorption bands appear (Figure 2a). The most important are highlighted in Figure 2b. The new absorption bands are located in the range of 3200–3500 cm^–1^ (OH and NH stretching vibrations), at 1656 cm^–1^ (NH bending vibrations) after the disappearance of the NH_2_ absorption bands from Jeff units (1582 cm^–1^, Figure 2c), and by the decrease in the intensity of oxirane ring vibrations (903 cm^–1^, Figure 2d). Therefore, the FTIR spectra provide compelling evidence for the successful ring-opening polymerization. The analyses of the off-stoichiometric compositions were similar to that of the 1:1 PLCO:Jeff composition. The spectra of both 1:1 DGEBA:Jeff and 1:1 DGEBA:Cray cured compositions, compared to pure DGEBA, are provided as reference data in the Supporting Information (Figure S3).

The degree of crosslinking can be estimated by the determination of the gel content. In this way, the swelling ratio and percent extract of the most relevant compositions of PLCO thermoset polymers were calculated by using eqs 2 and 3, respectively. As noted in Table 1, low swell ratios (∼1.5) indicate a high degree of crosslinking, which correspond to a high molecular weight between crosslinks. Thus, in this case, more tightly bound structures are present. Moreover, low values of percent extract (2–5%) also corroborate to a high degree of crosslinking. The stoichiometric compositions presented values of swelling ratio in xylene at 80 °C close to 1.5, whereas non-stoichiometric compositions varied from 1.5 to 1.7. From the percent extracts, it is possible to certify that all mixtures are well cured, presenting values of ∼92–97% of gel content (% by mass of insoluble polymer). Summarizing, the pre-curing and post-curing thermal processes were efficient and both stoichiometric and sub-stoichiometric samples are efficiently crosslinked.

**Table 1:** Thermal Properties, VOC Content, and Gel Content of the Main Epoxy:Hardener Compositions Prepared in the Present Work, with Variable Molar Ratios^a^

		DSC data				TGA data			gel content^3^	
epoxy:hardener	molar ratio	*T*_g∞_ (°C)	Δ*C*_p_ (J/g·K)	*T*_curing_ (°C)	*t*^1^ (min)	*T*_d_^5%^ (°C)	*T*_d,max_ (°C)	VOC (g/L)	swell ratio	percent extract (%)
PLCO:Jeff	1:1	62	0.192	142	30	230	258, 365	441	1.47 ± 0.04	2.74 ± 0.54
PLCO:Jeff	1:2	12	0.414	141	37	241	257, 354	297	1.46 ± 0.08	5.29 ± 0.37
PLCO:Cray	1:1	18	0.448	129	41	207	228, 419	524	1.53 ± 0.02	5.76 ± 1.02
PLCO:Cray	2:1	26	0.313	135	59	210	230, 406	602	1.67 ± 0.04	8.36 ± 1.34
PLCO:Jeff:1-MI	1:1	51	0.358	139	42	225	258, 365	429		
PLCO:Cray:1-MI	1:1	38	0.212	148	35	205	228, 354	512		
DGEBA:Jeff	1:1	30	0.421	112		326	377	400		
DGEBA:Cray	1:1	75	0.430	RT^2^	44	300	373, 433	503		
DGEBA:Cray	2:1	104	0.066	RT^2^	11	295	370, 428	372		

a Notes: ^1^time to reach 100% of degree of conversion; ^2^room temperature, ^3^ASTM 2765-16.

### Thermal Properties of PLCO:Hardener Compositions

From the dynamic DSC assays, it was possible to determine the curing temperatures and the ultimate glass-transition temperature (*T*_g∞_) of the new thermoset materials (Figures 3 and S4). Two heating processes were performed, and the second heating curves were used for determining the *T*_g∞_ (Table 1 and Figures S5 and S6). The thermal properties varied significantly comparing PLCO and DGEBA compositions and well depended on the stoichiometry of epoxy:hardener mixtures.

The *T*_g∞_ values indicate that upon heating, the polymer chain mobility can be easily controlled by varying the curing agent and the ratio of PLCO:hardener composition. For example, a 1:1 PLCO:Jeff ratio renders more rigid polymers (*T*_g∞_ 62 °C) at room temperature (r.t.) compared to a 1:2 PLCO:Jeff composition (*T*_g∞_ 12 °C) and a 1:1 DGEBA:Jeff composition (*T*_g∞_ 30 °C). Therefore, the constrained chemical structure of PLCs is balanced with the amine agent to offer thermoset materials with modulated *T*_g_s.^25^ Then, enhancing the Jeff content leads to a sharp decrease in the glass-transition temperature from 62 °C (PLCO:Jeff, 1:1) to 12 °C (PLCO:Jeff, 1:2), thus giving rise to more flexible thermoset chains at ambient temperature. On the contrary, when 1 equiv of Cray is used as a hardener, the resultant polymer (i.e., PLCO:Cray 1:1) requires a similar time curing to PLCO:Jeff 1:2 to reach 100% epoxy conversion (i.e., 41 min and 37 min, respectively, Table 1), as determined by isothermal DSC (Figure 3b). For the isothermal tests, the samples were completely cured during 2 h at constant temperatures shown in parentheses in the legend of Figure 3b. After evaluation of the first heating scans in the dynamic DSC curves, at different heating rates (data not shown), it was possible to determine that the compositions with Jeffamine demand slightly higher curing temperatures (165 °C) than Crayamid (160 °C). The curing time for full conversion of the polymer through the curing period is shown in Table 1, and the curing degree of the assessed polymers is contrasted in Figure 3b.

Overall, PLCO is more reactive toward polyetheramines (Jeff), and a stoichiometric ratio between these two components resulted in complete curing after 30 min without the need for a catalyst or an initiator molecule (Table 1). The addition of the curing accelerator (1-MI) did not improve the curing conversion time of the 1:1 PLCO:Jeff composition, whereas the curing time for the 1:1 PLCO:Cray mixture containing 1-MI decreased to a factor of 0.85 with respect to the same composition without the initiator. Thus, no significant improvement was observed by using 1-MI, and therefore, it was omitted in the subsequent preparation of thermoset films for further tests.

It is important to emphasize that the DGEBA:Cray mixture was taken as an example of a good curing epoxy-amine material to compare the reactivity of PLCO. The shortest curing time observed for a 2:1 DGEBA:Cray mixture (11 min) was expected due to fast curing at high temperatures (160 °C). Particularly interesting is the fact that a 1:1 PLCO:Jeff composition can efficiently cure in only 30 min (100% conversion degree) after post-curing activation at 165 °C, whereas the lowest reactivity is obtained for a 2:1 PLCO:Cray combination (59 min). According to Figure 3b and Table 1, the calorimetric studies illustrate a higher reactivity for the Jeff-containing compositions than for the Cray-based thermosets as complete curing was achieved after shorter periods.

The reactivity of DGEBA with Jeff is clearly higher than that of PLCO, as can be observed in Figure S6a when compared to Figure S5a, with a clear glassy to rubbery jump (high heat capacity, Δ*C*_p_). However, the lower reactivity of PLCO is not a limitation to obtain good polymeric films once the necessary curing processes have been applied. The high temperatures employed for its curing are usually applied in epoxy coating formulations used as stoving enamels, for example, in the automotive industry. Moreover, PLCO:Jeff and PLCO:Cray combinations are thermally stable with degradation temperatures starting at 200–230 °C (*T*_d_^5%^), whereas the maximum peaks are observed at 220–250 °C (*T*_d,max_) (Figure 3c,d, Table 1). Unfortunately, such values are about 100 °C lower than that observed for DGEBA:Jeff (1:1) and DGEBA:Cray (1:1) (Figure S6d, Table 1), proving that PLCO-based thermosets are less stable than DGEBA-based ones. Moreover, the lower *T*_d_^5%^ achieved with Cray as a hardener (even in PLCO or in DGEBA mixtures) can be attributed to its higher molecular weight compared to Jeff (i.e., higher AHEW). Too long macromolecules with steric hindrance can hinder the epoxy reaction.

The stress–strain tests were performed to evaluate the mechanical behavior of the new biobased systems, and their resistance to electrolyte penetration was assessed by means of EIS experiments (vide infra).

### Mechanical Behavior and Permeability of Films Composed of PLCO Biobased Epoxy

**Figure 4:**
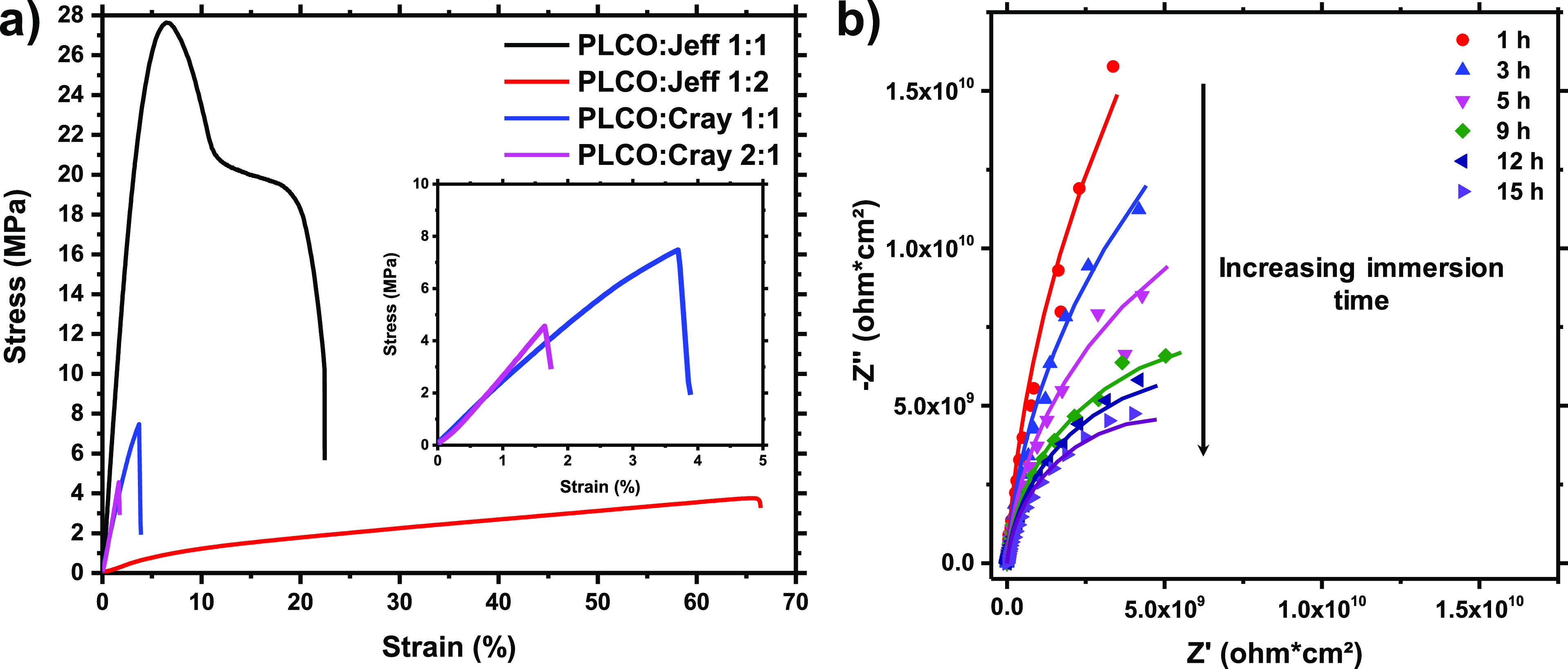
(a) Stress–strain curves of PLCO:Jeff (1:1 and 1:2) and PLCO:Cray (1:1 and 2:1); (b) Nyquist plots of PLCO:Jeff (1:1) films adhered to aluminum, with increasing immersion time in NaCl 0.05 M solution. Symbols correspond to the experimental results, and lines correspond to the fitted EEC.

As can be seen in Figure 4a, the stress–strain behavior of the PLCO thermoset materials varied a lot with the two different hardeners and with the stoichiometry of biobased epoxy:curing agents. The maximum tensile strengths at break found for 1:1 and 1:2 PLCO:Jeff mixtures were 27.5 ± 2.3 MPa and 3.9 ± 1.5 MPa, respectively, whereas the maximum elongations at break were 21.4 ± 4.9% and 68.0 ± 8.8%, respectively (Table S3). Such excess of curing agent Jeff, composed of mobile ether and methylene groups, imparts flexibility to the material, and this excess results in less crosslinked material; that is, PLCO:Jeff 1:2 has a gel content of 94.7% compared to 97.3% obtained with a 1:1 composition. Moreover, its rubber performance in stress–strain measurements can be predicted due to its low *T*_g_ (12 °C), below room temperature. On the other hand, Cray hardeners offered thermoset films with much more brittle characteristics (Figure 4a, inset) than Jeff, with low tensile strength and elongation at break (Table S3). The major difference in the mechanical performance is due to the chemical structure of the Cray commercial hardener. Either the formation of hydrogen bonds (promoted by amide groups) or the presence of aromatic groups (proved by FTIR, Figure S7) can favor the rigidity of the polymer network.

To conclude, from the stress–strain measurements, it is possible to tune the mechanical behavior of biobased thermoset PLCO by combining different molar ratios of commercial hardeners. It is well known that an excess of hardener is associated with better substrate adhesion at the expense of solvent resistance.^42^ Therefore, compositions with twice the content of Jeff would be desirable for adhesive technologies or coil coating formulations, which need flexibility to enable the coated metal to be bent without cracking or loss of adhesion of the paint film. On the other hand, an excess of PLCO will impart tenacity and a high Young modulus to the thermoset films, which would be desirable for solvent-borne protective coatings, in stoichiometric formulations. As the present study intends to push forward the application of PLCO in solvent-borne paint formulations for metal protection, the 1:1 PLCO:Jeff composition (the highest Young’s modulus, tensile strength, and good elongation at break) was chosen for the electrolyte resistance experiments (Figure 4b). The choice for polyoxypropylenediamine (Jeff) against polyaminoamide (Cray) is also justified by its solvent-free nature and its potential to be used in eco-friendly coating preparations such as in powder coatings.

Figure 4b depicts the Nyquist plots from impedance analysis using polymeric films composed of PLCO:Jeff (1:1) well cured above aluminum alloy plates (AA2024). At any exposure time, a perfect semi-circle is envisaged, corresponding to a highly resistive film. As expected, the semi-circle decreases with increasing immersion times due to the penetration of the electrolyte toward the substrate interface. After 15 h, the coating resistance (*R*_c_) has been reduced by 1 order of magnitude only (from 10^11^ to 10^10^, Table S4), which represents a minimal loss of insulating properties of the film. Moreover, the non-ideal capacitance (CPE_c_, constant phase element), which is the parameter attributed to the surface reactivity, surface heterogeneity, and roughness related to current and potential distribution, remained very low (Table S4).^43^ From a qualitative point of view, the absence of a second semi-circle, which would be related to a second phase constant (σ), is a positive result. The appearance of other electrical phenomena at the metal surface, such as inductance or impedances with phase angle decay (Bode plots, not shown), is related to the presence of pitting and oxide formation on the aluminum surface.^43−45^ As the substrate was previously pre-treated with a zirconium oxide passivating layer^34,35^ similar to phosphatizing ones, the biobased thermoset is well adhered and the penetration of the liquid does not attack the metal surface in the time interval applied.

Overall, these results allow us to believe that a stoichiometric PLCO:Jeff composition is a promising candidate for coating and adhesive technologies. The electrical parameters from the EEC fitting can be consulted in Table S4.

### Solvent-Free Two-Component Partially Biobased Epoxy Paint Formulation

To test the compatibility of a PLCO biobased solid epoxy with a DGEBA liquid resin, a high-solid paint was prepared. Some studies have addressed the partial replacement of DGEBA resin by bioresins as a mean to transition toward a more sustainable epoxy technology while trying to improve thermal–mechanical behavior of the classical DGEBA-based thermoset.^42^ A complete substitution is still unpractical due to the high volumes of biobased feedstock required ranging from kilograms to tons.

The kind of high-solid bicomponent epoxy paste prepared in this work is typically used for self-leveling of building structures (for example, to coat mineral substrates), mortars, and concrete and also as an anticorrosion coating for steel structures, where thick films are required.^14^ It is also classified as a solvent-free coating because it is does not use VOCs in the paint formulation. As can be noted in the Experimental Section, we replaced xylene by benzyl alcohol, which has a higher boiling point (203–205 °C vs 137–140 °C) and is less hazardous according to the European Community Regulation (EC no 1272/2008).^46^ Moreover, the classification “solvent-free coating” is based on a low content of the solvent (<2 pbw), which is indispensable to prepare a homogeneous paste.

**Figure 5:**
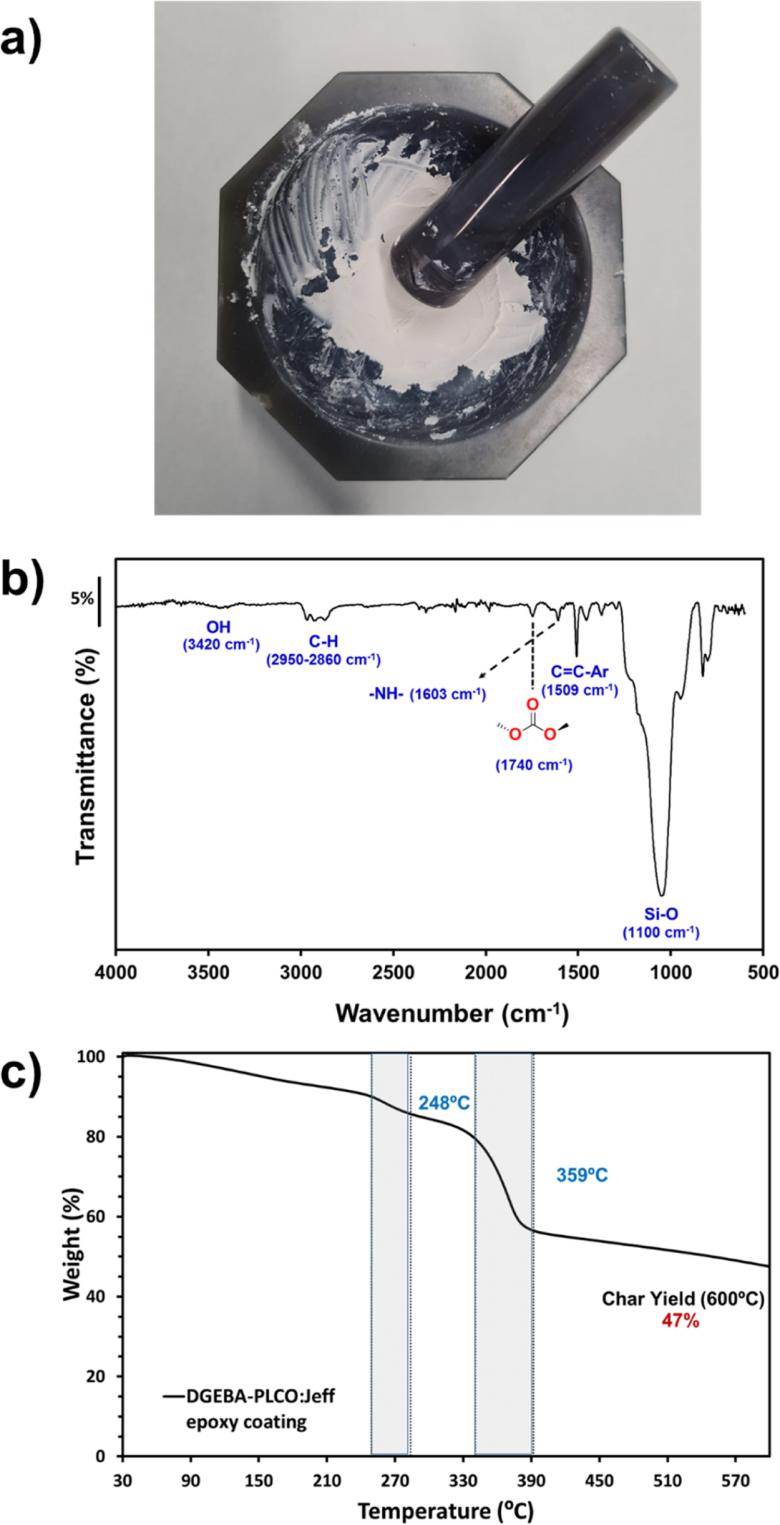
(a) Visual aspect of the high-solid two-component epoxy coating prepared with 20% of PLCO biobased epoxy and 80% of synthetic DGEBA, with Jeffamine as a curing agent, after mortar milling; (b) FTIR–ATR spectrum; and (c) TGA of the crosslinked coating.

The new coating was characterized by FTIR and TGA. Figure 5a displays the physical nature of the paste obtained after mortar milling, Figure 5b shows the chemical composition analyzed by FTIR, and Figure 5c shows the thermal stability of the solid film. The solid film obtained after post-curing treatment does not have the yellowish color shown in Figure 1c for the thermoset PLCO:Jeff-derived polymer. This observation is important if the product were to be applied, for example, for mortar or cement, which are white in color. FTIR shows the main absorption bands from the PLCO component (the main chain linear carbonate group at 1740 cm^–1^) and from DGEBA (aromatic C=C stretching bands at 1500 cm^–1^) as well as hydroxyl (3420 cm^–1^) and amine absorption bands (1600 cm^–1^). However, the strongest and sharpest absorption bands in Figure 5b belong to the filler (Si–O, 1230–945 cm^–1^), which is usually added in a high content to this epoxy paint.^47^

The thermal stability of the new DGEBA-PLCO:Jeff-based coating is much inferior to that of the pure films composed of 1:1 PLCO:Jeff or 1:1 DGEBA:Jeff. The materials starts to degrade (*T*_d_^5%^) at 150 °C. There are two degradation steps, one at 248 °C and the second and most prominent one at 359 °C (Figure 5c). The first decomposition is attributed to the PLCO content, and it is proportional to the amount added in the paste formulation. The second step can be assigned to the DGEBA-PLCO blend because it coincides with the *T*_d,max_ observed for PLCO:Jeff (1:1) and is slightly inferior to the pure 1:1 DGEBA:Jeff thermoset composition. The high-solid content is evidenced by the char yield at 600 °C (47%), which corresponds to the sum of TiO_2_ and the filler (Si NPs).

## Conclusions

For the first time, a biobased PLCO with high molecular weight and EEW values was combined with two synthetic hardeners to prepare a novel partially biosourced epoxy thermoset. The main advantage of the novel polymer is the ease of modulation of its thermal and mechanical properties by varying the molar ratio between the PLCO resin and the hardener content. The maximum tensile strength was found for a 1:1 PLCO:Jeff composition having very good elongation at break, although the thermal degradation is slightly inferior for the biobased compositions compared to the DGEBA ones. Moreover, the results demonstrate that the globally used DGEBA pre-polymer is compatible with biosourced PLCO, providing a stable solvent-free paint paste and cured films with polyoxypropylenediamine (Jeff) as a hardener.

Considering that the advantage of low-molecular-weight DGEBA is mainly due its low temperature requirement in the curing process, the use of PLCO cannot possibly compete yet. Nonetheless, the latter can be applied for stoving enamel and high-temperature epoxy coatings. Ultimately, pro-active sourcing of more sustainable feedstock will stimulate the demand and implementation of biobased precursors. Undoubtedly, biocomponents such as PLCO derived from limonene and carbon dioxide will contribute to current and future environmental compliance trends aiming for a low carbon and VOC footprint in coating technology.

## Supporting Information Available

Analytical and experimental data for raw materials and proportions of components in the studied compositions; solubility tests; FTIR spectra of cured films; DSC; TGA; mechanical properties; EIS equivalent-circuit data; and solvent-free coating formulation (PDF)
