# Supplementary material for: Novel Biobased Epoxy Thermosets and Coatings from Poly(limonene carbonate) Oxide and Synthetic Hardeners
Source: ACS Sustain Chem Eng. 2022 Feb 18;10(8):2708–19. doi: 10.1021/acssuschemeng.1c07665 (PMC8938888; doi:10.1021/acssuschemeng.1c07665)
Supplement: Supplementary file 1 — sc1c07665_si_001.pdf [file sc1c07665_si_001.pdf]

Supporting file

Novel biobased epoxy thermosets and coatings from poly(limonene carbonate) oxide and synthetic hardeners

Vitor Bonamigo Moreira,[1,2,3] Jeroen Rintjema,[4] Fernando Bravo,[4] Arjan W. Kleij,[4,5] Lourdes Franco,[1,3] Jordi Puiggalí,[1,3] Carlos Alemán,[1,3,6] Elaine Armelin[1,3]*

[1] Departament d'Enginyeria Química, Universitat Politècnica de Catalunya, Campus Diagonal Besòs (EEBE), C/ Eduard Maristany, 10-14, Building I, 2nd floor, 08019, Barcelona, Spain.

[2] Programa de Pós-graduação em Engenharias de Minas, Metalúrgica e de Materiais (PPGE3M), Universidade Federal do Rio Grande do Sul (UFRGS), Av. Bento Gonçalves, 9500 – 91501-970, Porto Alegre, RS, Brazil.

[3] Barcelona Research Center for Multiscale Science and Engineering, Universitat Politècnica de Catalunya, Campus Diagonal Besòs (EEBE), C/ Eduard Maristany, 10-14, Building I, basement floor, 08019, Barcelona, Spain.

[4] Institute of Chemical Research of Catalonia (ICIQ), The Barcelona Institute of Science and Technology, Av. Països Catalans 16, 43007 Tarragona, Spain.

[5] Catalan Institute of Research and Advanced Studies (ICREA), Pg. Lluis Companys 23, 08010 Barcelona, Spain

[6] Institute for Bioengineering of Catalonia (IBEC), The Barcelona Institute of Science and Technology, Baldiri Reixac 10-12, 08028 Barcelona, Spain.

Corresponding author: elaine.armelin@upc.edu

SUPPORTING INFORMATION

11 pages, 7 figures, 5 tables

Table of Contents

Table S1.. Pag. S3

Table S2.. Pag. S3

Figure S1.. Pag. S4

Figure S2.. Pag. S5

Figure S3.. Pag. S6

Figure S4.. Pag. S7

Figure S5.. Pag. S8

Figure S6.. Pag. S9

Figure S7.. Pag. S9

Table S3.. Pag. S10

Table S4.. Pag. S10

Table S5.. Pag. S11

EXPERIMENTAL SECTION

Table S1. Chemical data for the raw material used in the present work.

Raw material	Aspect	M_w [a)] (g/mol)	EEW [b)] (g/equiv)	AHEW [c)] (g/equiv)
**PLCO**	Powder	~ 8k-9k	216-315	-
**DGEBA**[d)]	Viscous Liquid	340.4	172-176	-
**DETA**[d)]	Liquid	103.5	-	21
**PEI**[d)]	Liquid	2000	-	37
**Jeff**[d)]	Liquid	430	-	115
**Cray**[d)]	Liquid	-	-	240-270

Notes: [a)] Data acquired by gel permeation chromatography; [b)] calculated by titration in alcoholic KOH (ASTM D1652); [c)] obtained from supplier datasheet; [d)] information available from commercial datasheets.

Table S2. Proportions of the components used in the studied compositions.

Epoxy:Hardener	Molar ratio	Epoxy (mg)	Hardener[a)] (mg)	Solvent (µL)	Initiator (mg)
**PLCO:Jeff**	1:1	100	53.2	50	-
**PLCO:Jeff**	1:2	100	106.5	50	-
**PLCO:Cray**	1:1	100	111.0	50	-
**PLCO:Cray**	2:1	100	55.5	50	-
**PLCO:DETA**	1:1	100	9.7	50	-
**PLCO:PEI**	1:1	100	17.1	50	-
**PLCO:Jeff:1-MI**	1:1	100	53.2	50	2.9
**PLCO:Cray:1-MI**	1:1	100	106.5	50	3.9
**DGEBA:Jeff**	1:1	100	66.9	50	-
**DGEBA:Cray**	1:1	100	139.5	50	-

Note: [a)] Stoichiometric amine content based on EEW = 216 g/equiv of PLCO and 172 g/equiv of DGEBA.

Gel permeation chromatography analysis

Gel permeation chromatography analyses were performed in THF solution at 40 °C at a flow rate of 1 mL/min and sample concentration of 1 g/L, after filtration through a 0.45 µm pore-size membrane. The separation was carried out on three polystyrene/divinylbenzene columns (Agilent, model PLgel 5µm MIXED-C, 300 × 7.5 mm). The setup (Viscotek TDA305) was equipped with a refractive index detector (λ = 670 nm). Data of M_n, M_w and M_w/M_n (PDI, *Đ*) were derived from the refractive index signal by a calibration curve based on polystyrene standards (from Polymer Standards Service).

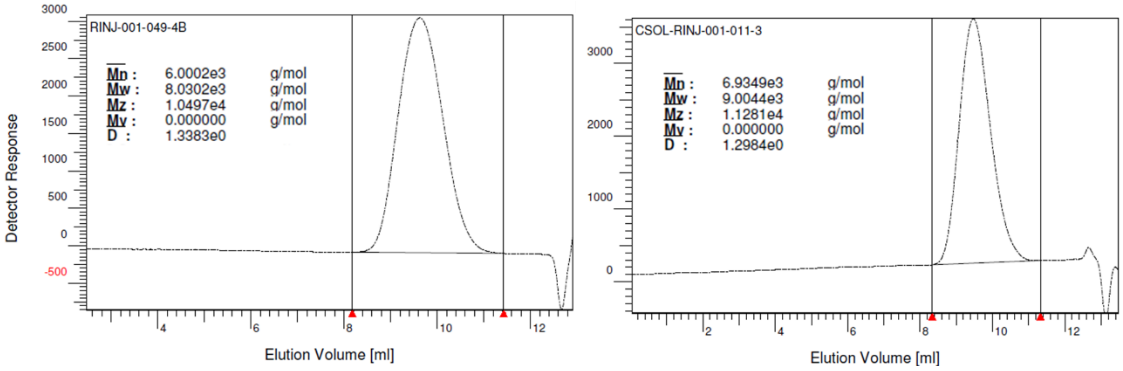

Figure S1. GPC curves of PLCO from two different batches.

RESULTS AND DISCUSSION

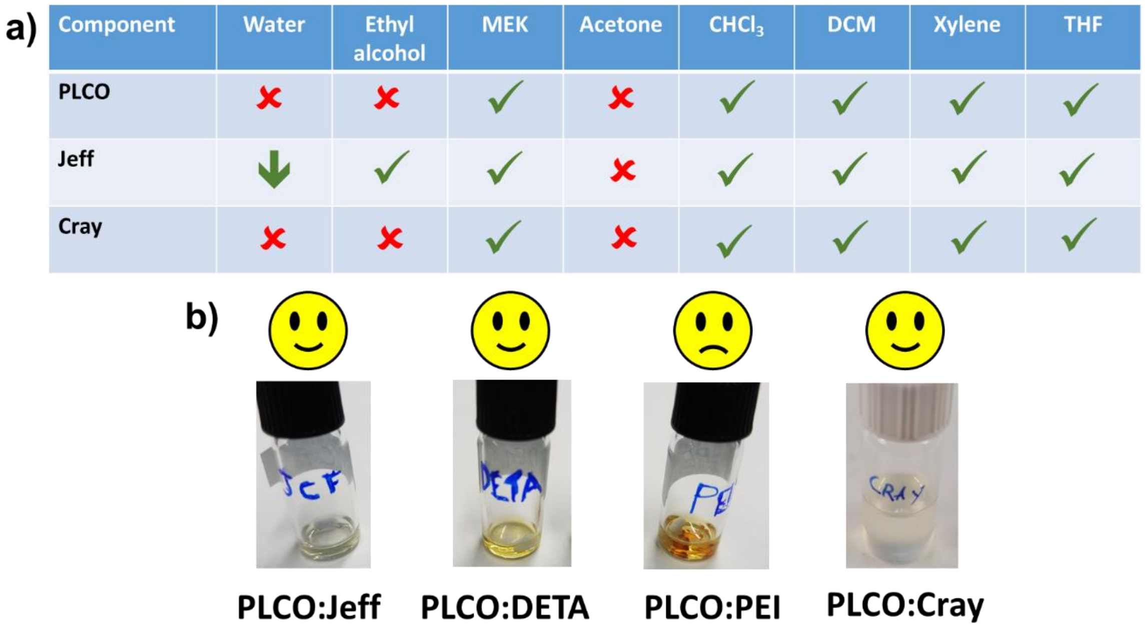

a)

Component	Water	Ethyl alcohol	MEK	Acetone	$CHCl_3$	DCM	Xylene	THF
PLCO	✗	✗	✓	✗	✓	✓	✓	✓
Jeff	↓	✓	✓	✗	✓	✓	✓	✓
Cray	✗	✗	✓	✗	✓	✓	✓	✓

Figure S2. (a) Solubility of PLCO and curing agents, separately, in most common polar solvents (MEK, methyl ethyl ketone; DCM, dichloromethane; THF, tetrahydrofuran), some of them used in coatings and inks technologies (ethyl alcohol, MEK, xylene). Symbols: (✗) insoluble, (↓) low solubility, (✓) soluble. (b) Visual aspect of the solution after mixture of PLCO and curing agents in 100 μL of xylene, at room temperature.

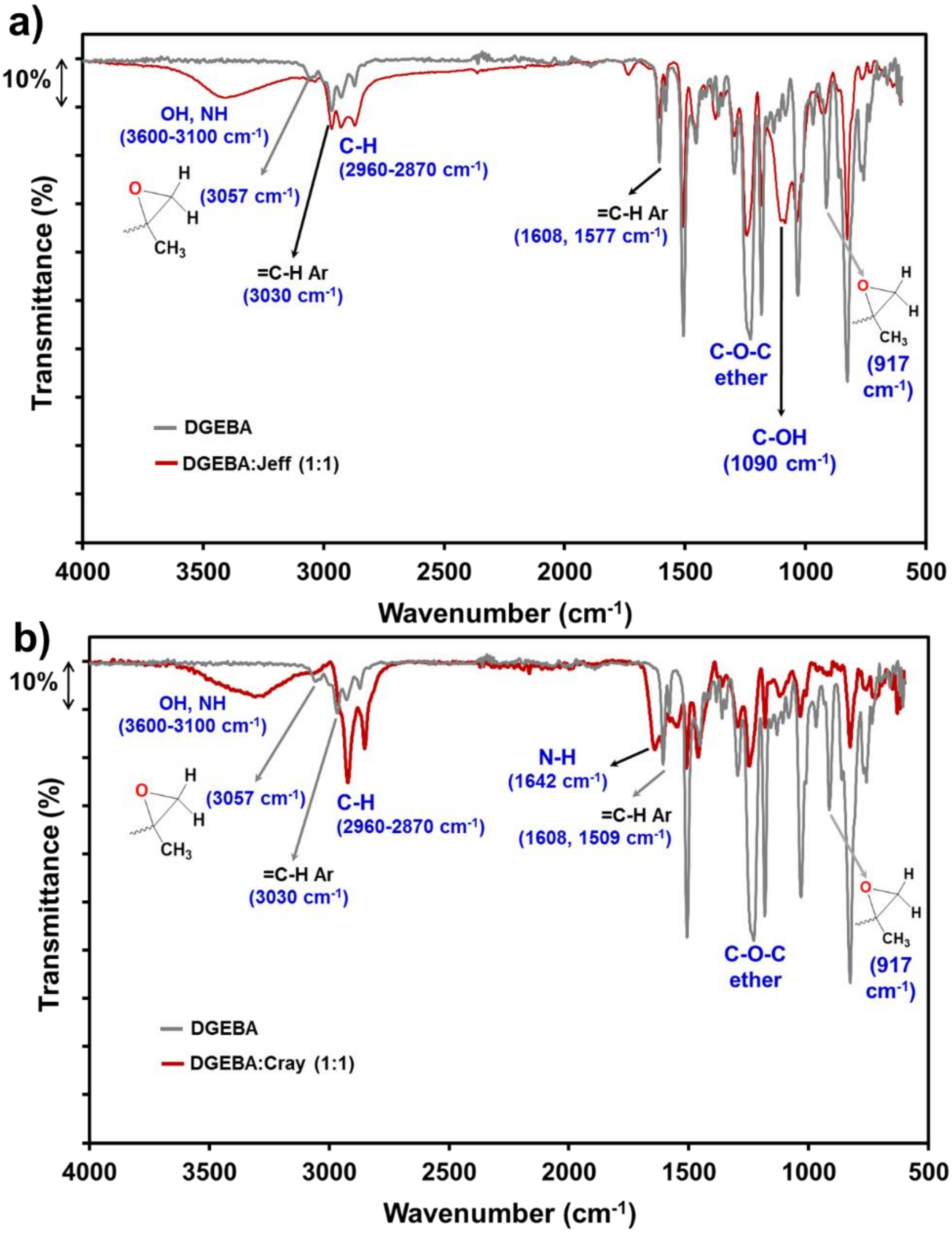

Figure S3. FTIR spectra of: (a) DGEBA:Jeff 1:1 and (b) DGEBA:Cray 1:1 cured films (r.t.), both compared to pure DGEBA prepolymer.

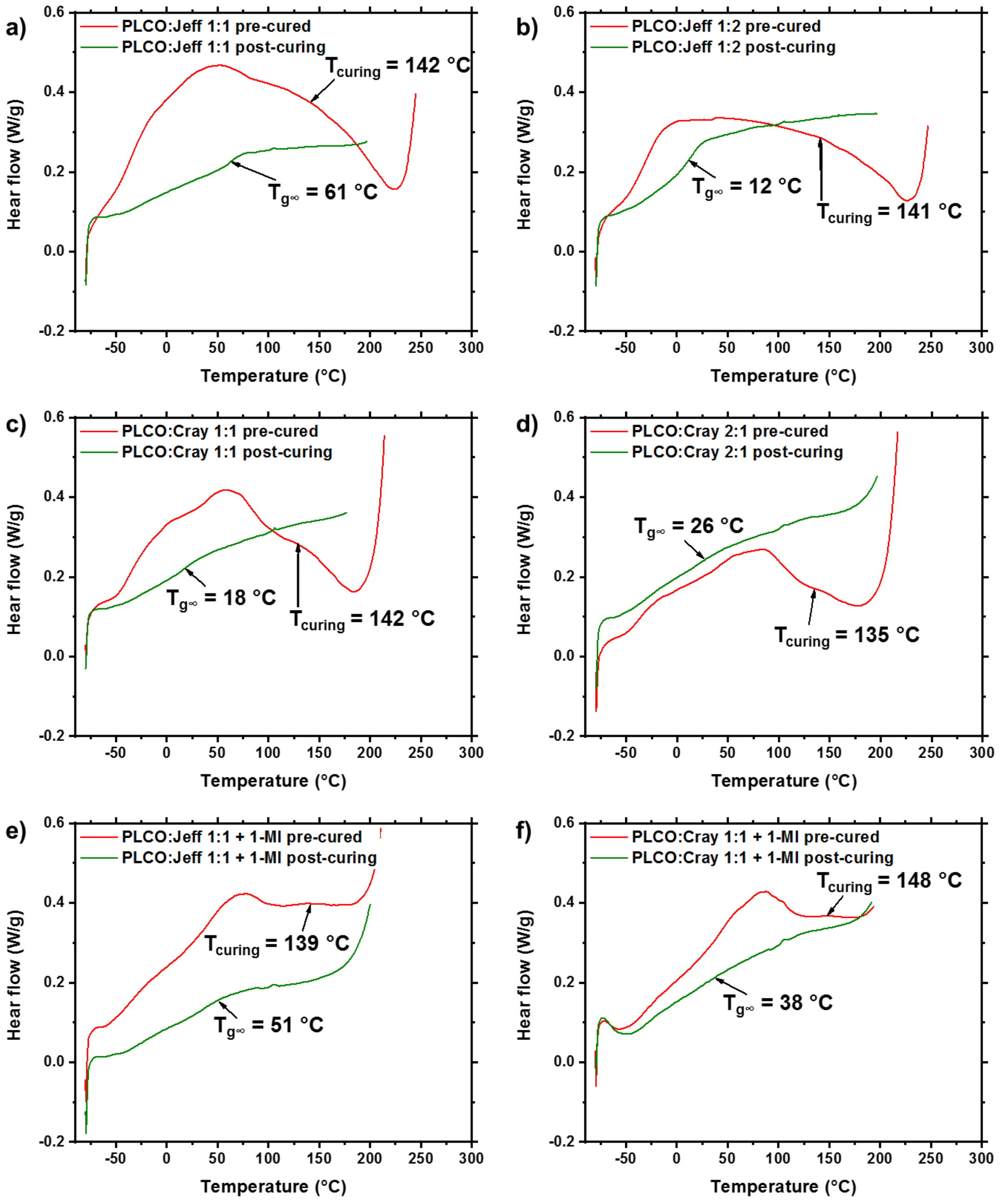

Figure S4. DSC first (red color) and second heating curves (green color) for pre-cured biobased thermoset films: (a) PLCO:Jeff 1:1; (b) PLCO:Jeff 1:2; (c) PLCO:Cray 1:1; (d) PLCO:Cray 2:1; (e) PLCO:Jeff 1-MI 1:1; and (f) PLCO:Cray 1-MI 1:1

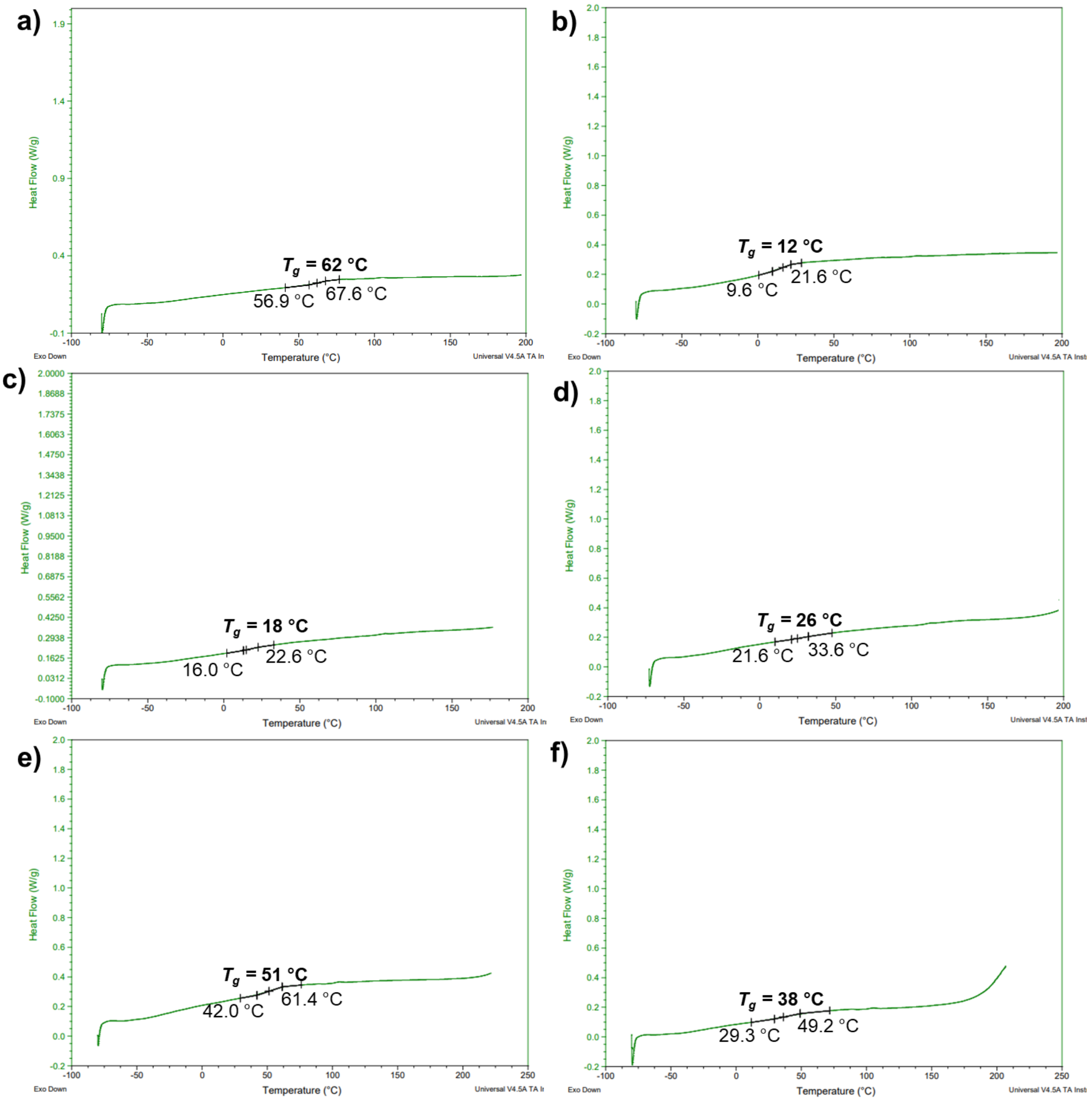

Figure S5. DSC second heating curves showing the T_g obtained for: (a) PLCO:Jeff 1:1; (b) PLCO:Jeff 1:2; (c) PLCO:Cray 1:1; (d) PLCO:Cray 2:1; (e) PLCO:Jeff 1-MI 1:1; and (f) PLCO:Cray 1-MI 1:1.

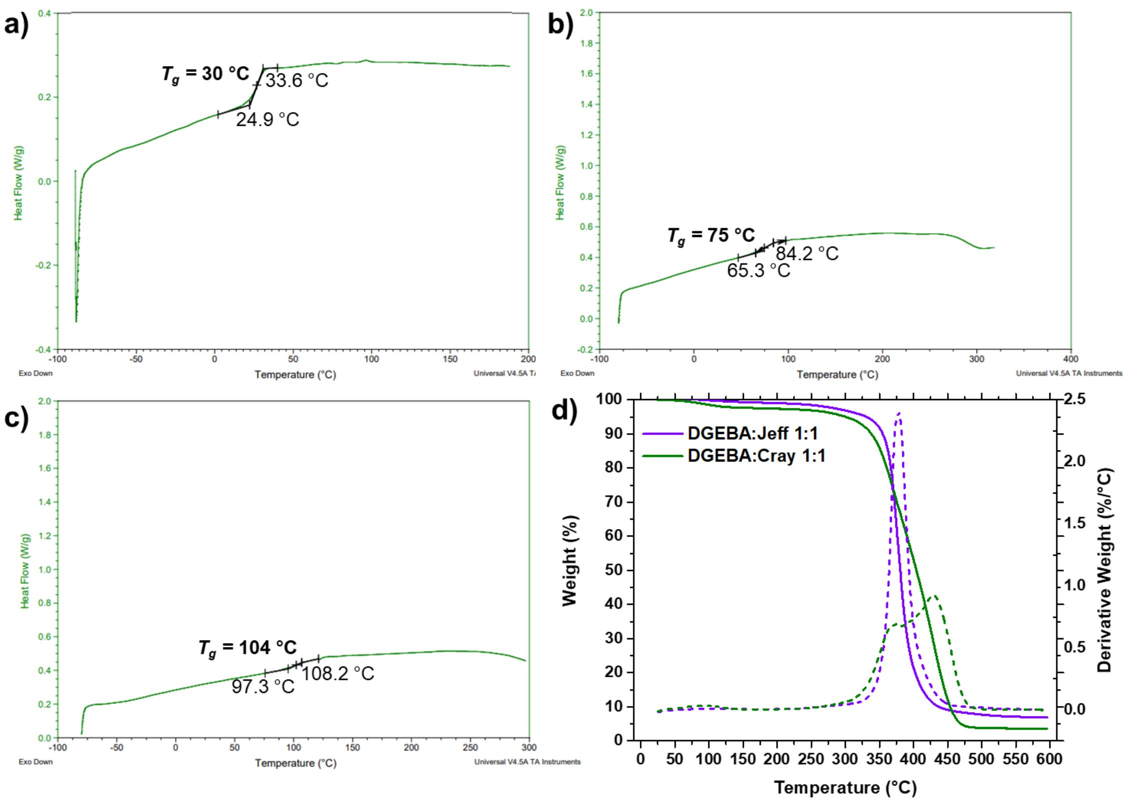

Figure S6. DSC second heating curves showing the T_g obtained for: (a) DGEBA:Jeff 1:1; (b) DGEBA:Cray 1:1; (c) DGEBA:Cray 2:1, and TGA analyses of: (d) DGEBA:Jeff 1:1 and DGEBA:Cray 1:1 cured samples.

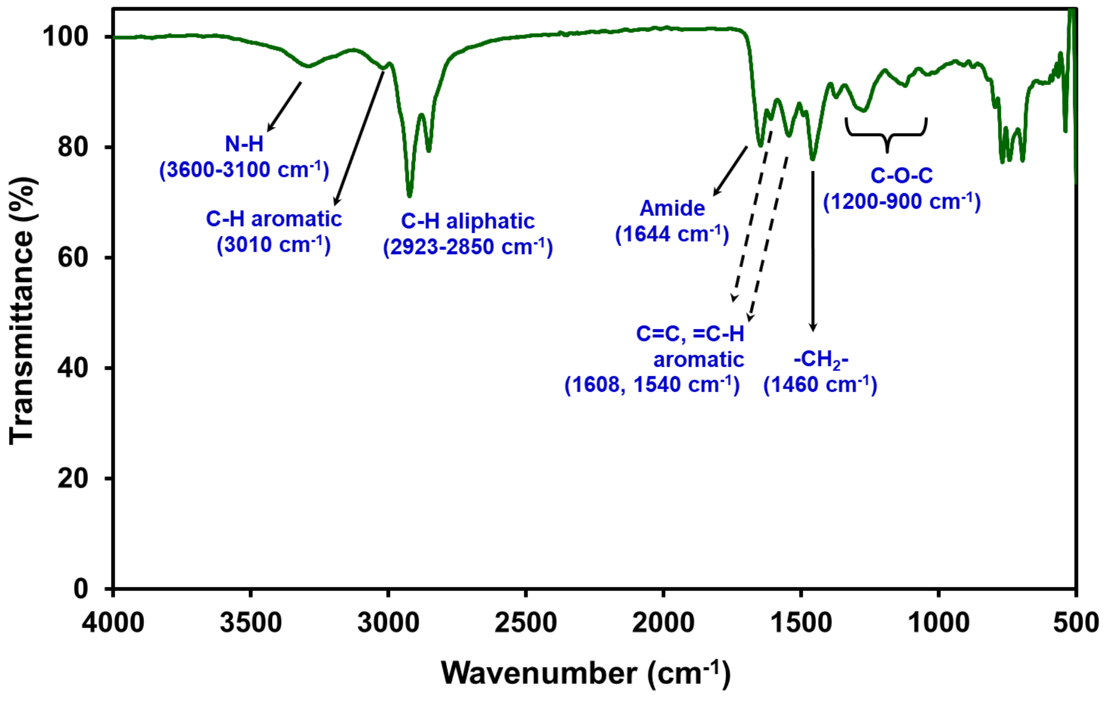

Figure S7. FTIR spectrum of Crayamid curing agent.

Table S3. Mechanical properties for the thermoset films prepared with PLCO and polyetheramine (Jeff) and polyamineamide (Cray), as curing agents.

Epoxy:Hardener	Molar ratio	σ_{max} (MPa)	Young modulus (MPa)	ε_{break} (%)
**PLCO:Jeff**	1:1	27.5 ± 2.3	523.0 ± 99.2	21.4 ± 4.9
**PLCO:Jeff**	1:2	3.9 ± 1.5	-	68.0 ± 8.8
**PLCO:Cray**	1:1	7.2 ± 1.2	209.5 ± 14.7	3.5 ± 0.5
**PLCO:Cray**	2:1	4.1 ± 0.7	282.2 ± 7.5	1.5 ± 0.2

Table S4. Data of EIS results obtained from the equivalent circuit for PLCO:Jeff (1:1) films, after exposure to NaCl aqueous solution (0.05 M).

Sample code	Exposure time (h)	R_s ($\Omega\cdot cm^2$)	R_c ($\Omega\cdot cm^2$)	CPE_c ($F\cdot cm^{-2}\cdot s^{n-1}$)	n_{CPE}
**PLCO:Jeff (1:1)**	1	368	1.13×10^{11}	5.03×10^{-11}	0.96
	3	321	5.79×10^{10}	5.80×10^{-11}	0.96
	5	390	3.35×10^{10}	6.49×10^{-11}	0.95
	9	345	1.92×10^{10}	7.02×10^{-11}	0.95
	12	340	1.65×10^{10}	8.19×10^{-11}	0.94
	15	366	1.28×10^{10}	8.32×10^{-11}	0.94

Table S5. Solvent-free two-component partially biobased epoxy paint formulation.

Position	Raw material	Function	Weight %
**Component A**			
**01**	PLCO	Biobased epoxy	8.46
**02**	DGEBA	Synthetic epoxy	33.82
**03**	TiO_2	Pigment	5.01
**04**	SiNPs	Filler	48.67
**05**	BYK A530	Defoamer	2.02[a)]
**06**	Benzyl alcohol	Solvent	2.02[a)]
**Sum A**			100
**Component B (hardener)**			
**07**	Jeffamine D400 [b)]	Curing agent	27.11[c)]

Notes: [a)] Calculated by density conversion (0.990 g/cm^3 and 0.972 g/cm^3, defoamer and solvent, respectively); [b)] Density: 0.972 g/cm^3; [c)] Stoichiometric and off-stoichiometric amine content based on EEW = 216 g/equiv of PLCO and 172 g/equiv of DGEBA.
